# SiteBinder – an improved approach for comparing multiple protein structural motifs. Case studies on biologically important motifs

**DOI:** 10.1186/1758-2946-4-S1-P59

**Published:** 2012-05-01

**Authors:** David Sehnal, Radka Svobodová Vařeková, Heinrich J Huber, Stanislav Geidl, Crina-Maria Ionescu, Michaela Wimmerová, Jaroslav Koča

**Affiliations:** 1 National Centre for Biomolecular Research and CEITEC - Central European Institute of Technology, Masaryk University, Brno, Czech Republic, 625 00, CZ; 2 Systems Biology Group, Royal College of Surgeons in Ireland, Dublin, Ireland, D2, IE

## 

Novel high-throughput experimental techniques produce a large amount of data on the 3D structure of proteins and their structural motifs. These motifs can be used as patterns in drug discovery [1], can help to understand the relationship between a protein’s structure and its function [2] and to classify proteins [3]. In order to extract as much information as possible from this data, new techniques and tools are necessary, and among them fast approaches to perform the multiple superimposition of large sets of protein structural motifs. We report here on the development of such a tool.

We have implemented our newly developed multiple superimposition methodology in the web application SiteBinder, which is able to process hundreds of protein structural motifs in a very short time and provides an intuitive and user-friendly interface. We also demonstrate the applicability of SiteBinder using three case studies, focused on biologically important protein motifs. In the first case study, we compared the structures of 67 PA-IIL sugar binding sites containing 9 different sugars and we found that the sugar binding sites of PA-IIL and its mutants have a conserved structure despite their binding different sugars. The second case study focused on more than 300 zinc finger central motifs and revealed that the molecular structure in the vicinity of the Zn atom in Cys2His2 zinc fingers is highly conserved. In the last case study, we superimposed 12 BH3 domains from pro-apoptotic proteins, and found that there is a structural basis for the functional segregation of these proteins into activators and enablers.
